# Regulation of IDO Activity by Oxygen Supply: Inhibitory Effects on Antimicrobial and Immunoregulatory Functions

**DOI:** 10.1371/journal.pone.0063301

**Published:** 2013-05-13

**Authors:** Silvia K. Schmidt, Sebastian Ebel, Eric Keil, Claudia Woite, Joachim F. Ernst, Anika E. Benzin, Jan Rupp, Walter Däubener

**Affiliations:** 1 Institute of Medical Microbiology and Hospital Hygiene, Heinrich-Heine-University Düsseldorf, Düsseldorf, Germany; 2 Institute for Molecular Mycology, Heinrich-Heine-University Düsseldorf, Düsseldorf, Germany; 3 Institute of Medical Microbiology and Hygiene, University of Lübeck, Lübeck, Germany; 4 Medical Clinic III/UK-SH, Campus Lübeck, Lübeck, Germany; University of Colorado Denver, United States of America

## Abstract

Tryptophan is an essential amino acid for human beings as well as for some microorganisms. In human cells the interferon-γ (IFN-γ) inducible enzyme indoleamine 2,3-dioxygenase (IDO) reduces local tryptophan levels and is therefore able to mediate broad-spectrum effector functions: IDO activity restricts the growth of various clinically relevant pathogens such as bacteria, parasites and viruses. On the other hand, it has been observed that IDO has immunoregulatory functions as it efficiently controls the activation and survival of T-cells. Although these important effects have been analysed in much detail, they have been observed *in vitro* using cells cultured in the presence of 20% O_2_ (normoxia). Such high oxygen concentrations are not present *in vivo* especially within infected and inflamed tissues. We therefore analysed IDO-mediated effects under lower oxygen concentrations *in vitro* and observed that the function of IDO is substantially impaired in tumour cells as well as in native cells. Hypoxia led to reduced IDO expression and as a result to reduced production of kynurenine, the downstream product of tryptophan degradation. Consequently, effector functions of IDO were abrogated under hypoxic conditions: in different human cell lines such as tumour cells (glioblastoma, HeLa) but also in native cells (human foreskin fibroblasts; HFF) IDO lost the capacity to inhibit the growth of bacteria (*Staphylococcus aureus*), parasites (*Toxoplasma gondii*) or viruses (herpes simplex virus type 1). Additionally, IDO could no longer efficiently control the proliferation of T-cells that have been co-cultured with IDO expressing HFF cells *in vitro*. In conclusion, the potent antimicrobial as well as immunoregulatory functions of IDO were substantially impaired under hypoxic conditions that pathophysiologically occurs *in vivo*.

## Introduction

Human hosts are permanently confronted with a variety of pathogens that have the potency to infect every niche within the body. Therefore, efficient defense strategies of the host as well as immune escape mechanisms of different pathogens are constantly in the scope of research. One major antimicrobial effector mechanism is the tryptophan-degrading enzyme indoleamine 2,3-dioxygenase (IDO), which can be induced in many human cell types, including myeloid cells, fibroblasts, epithelial cells and tumour cells upon stimulation with the pro-inflammatory cytokine interferon-γ (IFN-γ) by JAK-STAT signalling. IDO belongs to the family of heme enzymes that catalize the oxidative cleavage of tryptophan and this IDO-mediated local tryptophan depletion limits the growth of various pathogens including bacteria (e.g. Staphylococci, Streptococci, Enterococci), viruses (e.g. herpes simplex virus, measles virus) and parasites (e.g. Toxoplasma, Neospora) [1]. Hence, IDO is an important molecule of the innate immune response that is directed against a broad range of pathogens. In addition, IDO has been shown to influence the adaptive immune response as well. In this context IDO acts immunoregulatory, not only by depleting local tryptophan levels and therefore modulating T-cell responses, but also by producing immunorelevant, T-cell inhibitory metabolites and by enzymatic and non-enzymatic mechanisms resulting in tolerance [2]–[5].

Although these divergent roles of IDO have been investigated in much detail, most of the results have been obtained by *in vitro* studies. For this purpose different freshly isolated IDO-expressing cells or cell lines have been incubated in a regular humidified incubator at 37°C and under 21% O_2_ corresponding to the oxygen content in the air. Even though this normoxic air is inhaled, *in vivo* oxygen concentrations are much lower. Physiological oxygen levels lie for the most part between 3 to 5% O_2_ and rarely exceed 12% O_2_, even in well-vascularized tissues [6]. In pathologically altered tissues like infected or cancerous organs oxygen contents drop even to levels below 1% and tissue foci are therefore referred to as “hypoxic environment” [7], [8]. Host cells have to adapt to these low oxygen conditions and must optimize their cell energetics and homeostasis, especially since many common pathogens proliferate readily in hypoxic environments where they additionally deprive infected cells of O_2_ [9].

Up to now IDO-mediated effects under hypoxic conditions were only analysed in an infection model with intracellular bacteria. In 2010 Roth *et al.* described that the IFN-γ-mediated antichlamydial properties were abrogated under low oxygen concentrations in human fallopian tube cells due to disturbances in JAK-STAT signalling. As the activity of the IFN-γ-induced IDO enzyme was also diminished the cells were unable to limit the growth of *Chlamydia trachomatis* [10].

In this work we focussed on the effect of hypoxia on the IDO-mediated defense against a broader range of pathogens. We found that the expression and activity of IDO was impaired by hypoxia in different tumour cells (86HG39 and HeLa cells) and native cells (HFF cells) and that the tryptophan-dependent growth-inhibition of three prototypic pathogens in these cells was compromised, resulting in an unhindered proliferation of extracellular bacteria (*Staphylococcus aureus*), the intracellular parasite *Toxoplasma gondii* and herpes simplex virus type 1. Additionally, we examined the role of oxygen on the function of IDO in the regulation of T-cell responses. IDO was no longer able to inhibit the proliferation of activated T-cells under hypoxia. Therefore, IDO-mediated effector functions depend on the environmental oxygen supply and we discuss cellular advantages and disadvantages that result from insufficient IDO activation.

## Results

### IDO Activity and Expression are Reduced Under Hypoxic Conditions

**Figure 1:**
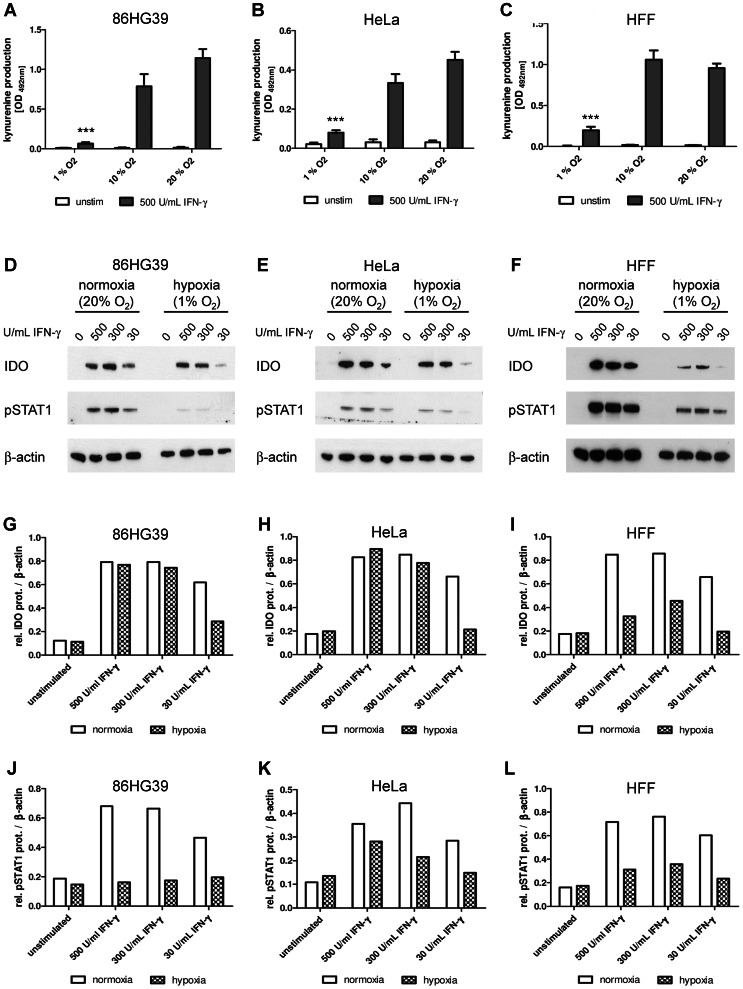
Reduced indoleamine 2,3-dioxygenase enzyme activity and expression under hypoxic conditions. (A–C) Determination of the kynurenine production in different cells after IDO induction by IFN-γ *in vitro*. Glioblastoma cells 86HG39 (A), HeLa cells (B) or human foreskin fibroblasts (HFF) (C) were stimulated with IFN-γ (500 U/mL) or left unstimulated in IMDM cell culture medium containing additional L-tryptophan (100 µg/mL). The 72 h incubation period was carried out at normoxia (20% O_2_), (10% O_2_) or hypoxia (1% O_2_). The kynurenine content in the cell culture supernatants was determined by optical density at 492 nm +/− SEM, using Ehrlichs reagent. A significant inhibition of kynurenine production as compared to the stimulated normoxia positive control is marked with asterisks, n = 3. (D-F) Western Blot analysis of 86HG39 (D), HeLa (E) or HFF lysates (F) stimulated with IFN-γ (0–500 U/mL) under normoxia or hypoxia for 24 h. Protein expression of IDO, phosphorylated STAT1 (pSTAT1) and β-actin was detected. (G-L) Densitometric analysis of the Western Blots shown in D–F. Relative protein expression of IDO and pSTAT1 with reference to the β-actin protein expression.

In order to disclose an impact of the local oxygen concentration on the enzyme activity of indoleamine 2,3-dioxygenase (IDO) the capacity of tumour and native cell lines to degrade tryptophan was analysed *in vitro*. Therefore IDO expression in human glioblastoma cells (86HG39), HeLa cells and human foreskin fibroblasts (HFF) was induced by treatment with IFN-γ (500 U/mL) and cells were incubated for 72 h under normoxic conditions (20% O_2_), intermediate oxygen conditions (10% O_2_) or under hypoxic conditions (1% O_2_), respectively. The enzyme activity of IDO was then determined by measuring the formation of the product kynurenine in the cell culture supernatants. As shown in figure 1 no kynurenine was detected in the supernatants of unstimulated cells but all cell lines produced kynurenine after stimulation with IFN-γ under normoxic conditions. This production of kynurenine was not impaired under intermediate oxygen conditions (10% O_2_) but it was significantly reduced under hypoxia (1% O_2_) (Fig. 1 A–C).

Since the reduction of kynurenine could result from a reduced IDO enzyme activity or from a reduced IDO expression we next analysed IDO protein levels in the different cell lines after a 24 h stimulation period with IFN-γ (0, 30, 300, 500 U/mL) under normoxia (20% O_2_) or hypoxia (1% O_2_) by Western Blotting. As expected, IDO protein was found after the stimulation with IFN-γ dose-dependently in all analysed cells. However, IDO protein levels were decreased upon hypoxia in all groups, suggesting that not only IDO activity but also IDO protein biosynthesis was diminished (Fig. 1D–F). Highly decreased IDO protein levels were especially detected under low stimulation conditions (30 U/mL IFN-γ) in tumour cells and in all HFF cell groups, where more than 60% of the protein was absent under hypoxia (Fig 1G–I).

Reduced IDO expression levels under low oxygen conditions can be a consequence of an impaired activation of the JAK-STAT signalling pathway [10]. In order to find out whether JAK-STAT signalling was also compromised by hypoxia in the analysed tumour and native cell models the amount of STAT1 protein was determined via immunoblotting. Under normoxic conditions the stimulation with IFN-γ-induced JAK-STAT signalling in all cells could be detected by the presence of phosphorylated STAT1 (pSTAT1). In contrast, pSTAT1 was nearly absent in tumour cells and was drastically reduced in HFF cells under hypoxia (Fig. 1D–F and 1J–L) indicating that JAK-STAT signalling was also affected in the *in vitro* models.

**Figure 2:**
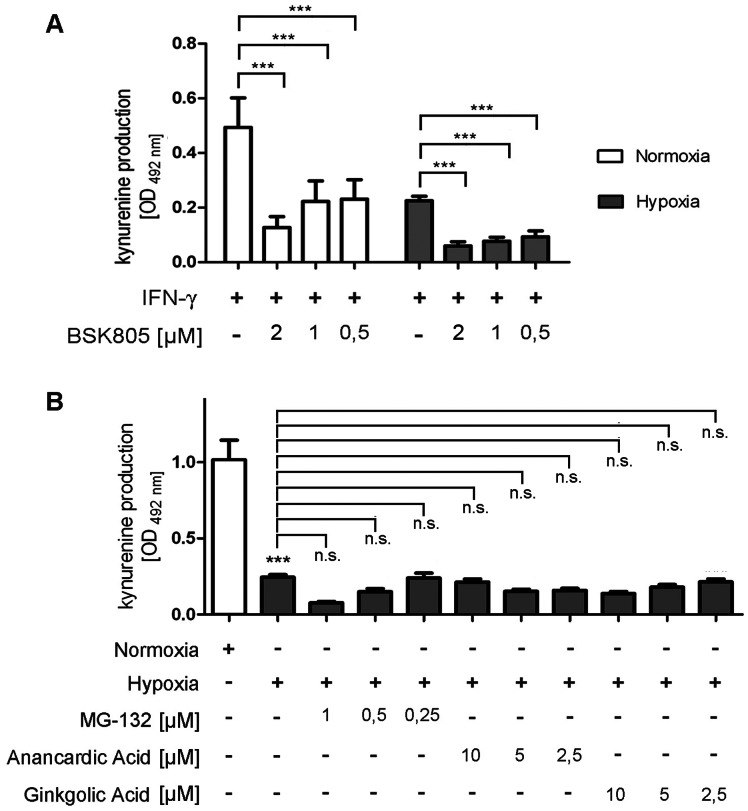
Inhibition of indoleamine 2,3-dioxygenase enzyme activity by different inhibitors. (A) Determination of the kynurenine production in HFF cells after IDO induction by IFN-γ (100 U/mL). The cells were incubated for 72 h under normoxia (20% O_2_) or hypoxia (1% O_2_) and treated with different amounts of the JAK2 inhibitor BSK805 (0–2 µM). (B) Kynurenine production of HFF cells after IDO induction by IFN-γ (100 U/mL). The cells have been incubated for 72 h under normoxia (20% O_2_) or hypoxia (1% O_2_) with different amounts of the proteasome inhibitor MG-132 (0–1 µM) or the sumoylation inhibitors Anancardic Acid (0–10 µM) or Ginkgolic Acid (0–10 µM). The kynurenine content in the cell culture supernatants was determined by optical density at 492 nm +/− SEM, using Ehrlichs reagent. A significant inhibition of kynurenine production as compared to the stimulated normoxia positive control is marked with asterisks. In B the addition of inhibitors did not result in a significant increase of the kynurenine production in cells incubated under hypoxia, this is marked by n.s. (not significant), n = 3.

To elucidate a possible influence of hypoxia on JAK2 we analysed the effectivity of the JAK2 inhibitor BSK805 on IFN-γ-induced IDO activity of HFF. As shown in figure 2A the addition of BSK805 to IFN-γ stimulated HFF reduces IDO activity. However the inhibitory effect was comparable under hypoxia and normoxia and reached 60–75% of the respective positive control. As shown in figure 1D–F the protein levels determined by the measurement of β-actin is slightly reduced in all probes from cells cultured under hypoxia. Therefore we excluded a potentially enhanced IDO degradation by the use of the proteasome inhibitor MG-132 and the sumoylation inhibitors Anancardic Acid or Ginkgolic Acid. As shown in figure 2B the addition of these inhibitors to IFN-γ stimulated HFF did not result in an enhancement of IDO activity in cells stimulated under hypoxia. We therefore conclude that enhanced protein degradation is not responsible for the observed IDO inhibition under hypoxic conditions.

**Figure 3:**
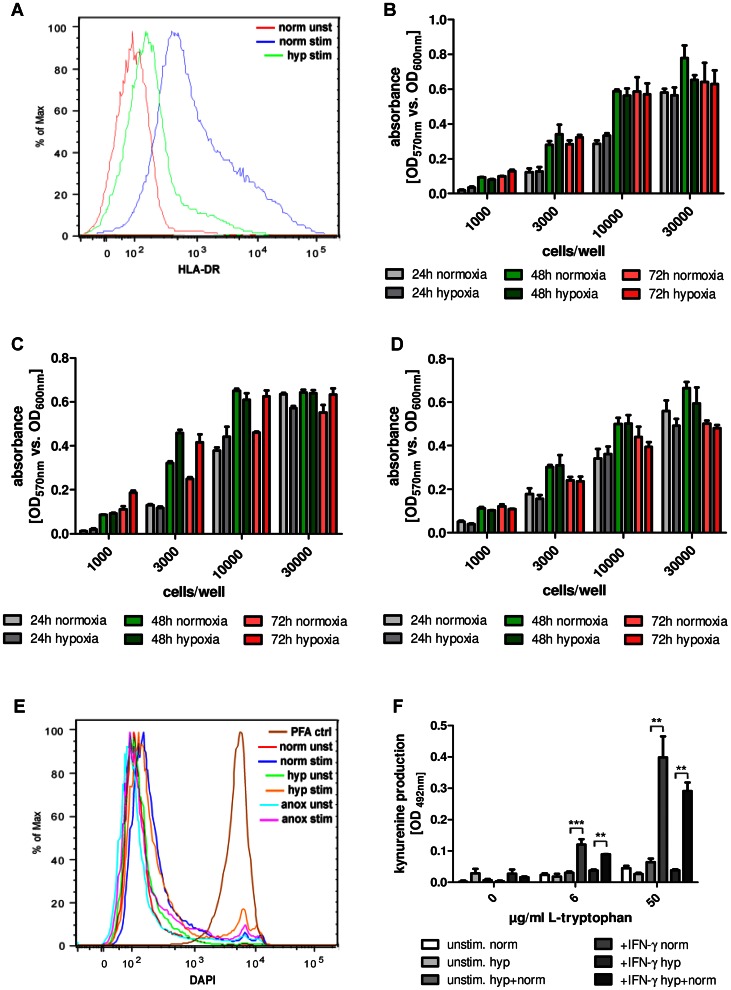
IFN-γ signalling and cell survival under different oxygen conditions. (A) FACS analysis of unstimulated or IFN-γ stimulated (100 U/mL) HeLa cells that have been incubated for 72 h under normoxia (20% O_2_) or hypoxia (1% O_2_) in cell culture medium with supplemental L-tryptophan (100 µg/mL). Cells were stained for HLA-DR. (B-D) Cell viability assay. Indicated cell numbers of 86HG39 glioblastoma cells (B), HeLa cells (C) or human foreskin fibroblasts (HFF; D) were incubated under normoxia or hypoxia (24–72 h). Then alamarBlue was added to the cells and the reducing power of living cells in the samples was measured quantitatively via absorbance at OD_570_/OD_600_+/− SD. Data of one representative experiment, performed in triplicates. (E) FACS analysis of unstimulated or IFN-γ stimulated (100 U/mL) HeLa cells, incubated for 72 h under normoxia, hypoxia or anoxia in cell culture medium with supplemental L-tryptophan (100 µg/mL). Cells were stained for DAPI as indicator for cell survival. PFA-fixed cells served as positive control for cell death. (F) Kynurenine amount in supernatants of unstimulated or IFN-γ stimulated (500 U/mL) HeLa cells in the presence of different amounts of L-tryptophan (0, 6 or 50 µg/mL). Cells were incubated for 72 h in normoxia or hypoxia and subsequently reoxygenated for 48 h in normoxia. Then the kynurenine content in the cell culture supernatants was determined by optical density at 492 nm +/− SEM, using Ehrlichs reagent. A significant inhibition of kynurenine production as compared to the stimulated normoxia positive control is marked with asterisks, n = 3.

JAK-STAT signalling is essential for the regulation of a broad palette of interferon-induced proteins [11]. We were therefore interested whether hypoxia also influences other IFN-γ induced and JAK-STAT dependent molecules and checked exemplarily the expression of HLA-DR, a cell surface receptor of the major histocompatibility complex (MHC) class II. For this purpose, HeLa cells were pre-stimulated with IFN-γ for three days, stained with DAPI and antibodies against human HLA-DR and analysed via FACS. Under normoxic conditions HeLa cells expressed HLA-DR after stimulation with IFN-γ as expected. In contrast, hypoxic incubation clearly decreased the expression of HLA-DR (Fig. 3A) demonstrating that hypoxia can indeed effectively interfere with the regulation of interferon-induced effector molecules as well.

Since not only IDO but also pSTAT1 and HLA-DR levels were decreased under hypoxic conditions, hypoxia-induced cell death had to be excluded. Cell survival was confirmed by several observations. First, there was only a slight difference in the protein levels of β-actin in normoxia- and hypoxia-cultivated cells in the Western Blot analysis (Fig. 1D–F). Second, cell viability assays based on alamarBlue reagent detected that both normoxia- and hypoxia-treated cells (24 h–72 h) had a comparable reducing power of resazurin which is a cell health indicator (Fig. 3B–D). Third, FACS analysis revealed that the bulk of cells survived at least the 72 h stimulation phase, not only under normoxia, but also under hypoxia and even under anoxia, whereas PFA fixed cells served as positive control for cell death (Fig. 3E). Fourth, IDO activity was restored in reoxygenated cells. Therefore, HeLa cells were stimulated with IFN-γ (500 U/mL) and incubated for an initial 72 h hypoxic phase, followed by a 48 h normoxic phase. Figure 3F shows that the cells efficiently produced kynurenine after reoxygenation and therefore confirms cell survival during the hypoxic phase.

Together these data indicate that the reduced IDO expression and activity were due to impairment in the activation of IFN-γ dependent JAK-STAT signalling by low oxygen conditions in tumour cells as well as in native cells but not the result of hypoxia-induced cell death.

### Antimicrobial Function of IDO is Abrogated Under Hypoxia

**Figure 4:**
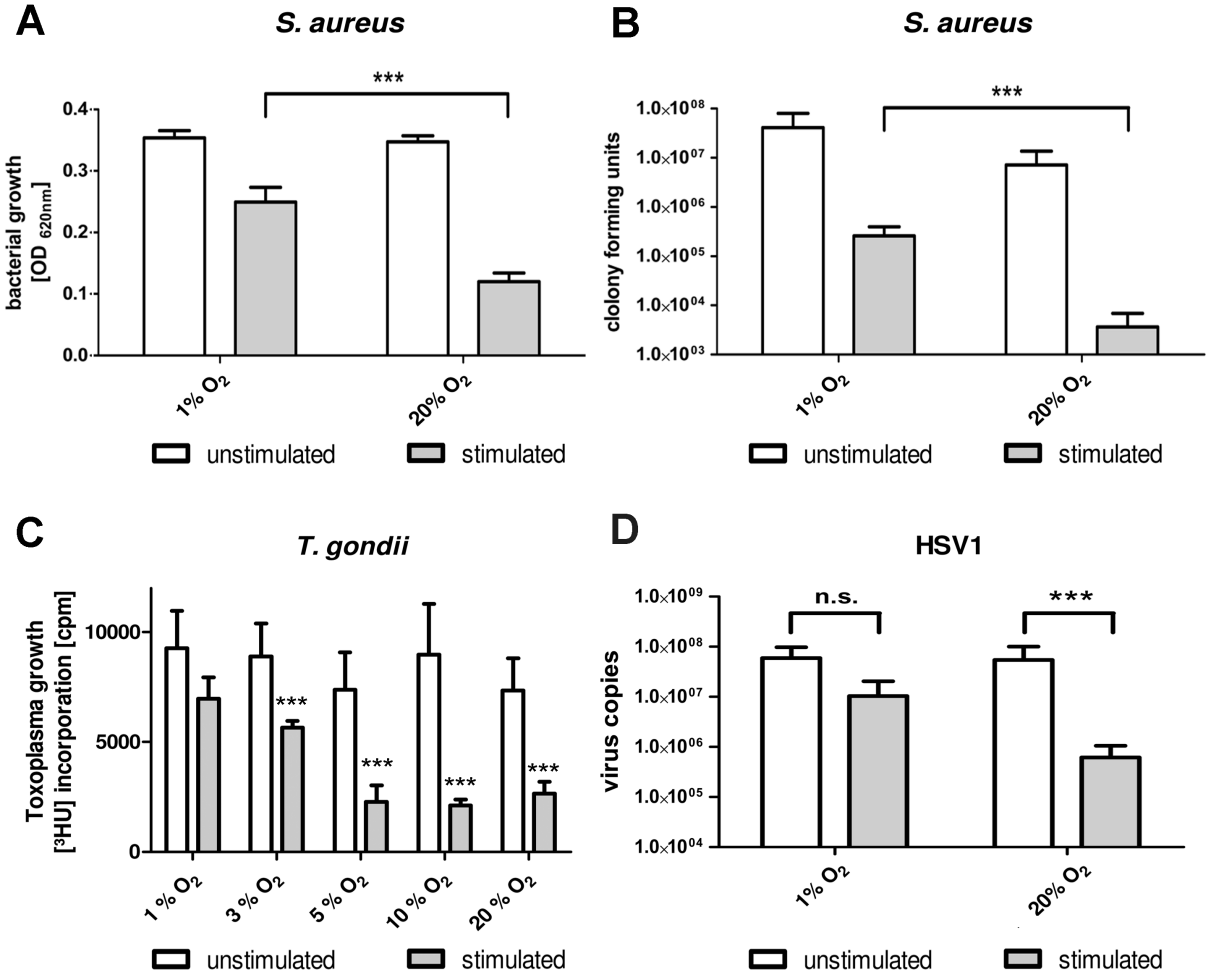
IDO-mediated antimicrobial effect is lost under low oxygen concentrations. (A) Growth of *Staphylococcus aureus* in supernatants of unstimulated or IFN-γ stimulated (500 U/mL) human fibroblasts, incubated for 72 h under different oxygen concentrations (1%–20% O_2_). Bacterial growth was determined 24 h after infection by optical density at 620 nm +/− SEM. (B) Proliferation of *Toxoplasma gondii* in HFF cells that have been pre-stimulated with 500 U/mL IFN-γ or not for 72 h under different oxygen concentrations (1%–20% O_2_). Then the cells were infected with the parasites and the Toxoplasma proliferation was determined after 48 h by the incorporation of ^3^H-uracil. (C) Replication of herpes simplex virus type 1 (HSV1) in pre-stimulated (500 U/mL IFN-γ or not) HFF cells that have been incubated for 72 h under normoxia (20% O_2_) or hypoxia (1% O_2_). After pre-stimulation cells were infected with HSV1 and viral replication was detected after additional 72 h via real-time PCR. A significant inhibition of bacterial, parasitic and viral growth, respectively, as compared to the stimulated normoxia positive control is marked with an asterisk (*), n = 3.

Next, we investigated whether hypoxia abolishes functional effects of IDO *in vitro*. Since IDO is well-known for its antimicrobial properties the IDO-mediated antibacterial, antiparasitic and antiviral functions within hypoxic environment are of particular interest. In different *in vitro* infection models it could be shown that an IFN-γ induced IDO activity was sufficient to control the growth of different microorganisms within tumour and native human cells [1]. Figure 4 shows the result of experiments using HFF cells that were infected with *Staphylococcus aureus*, *Toxoplasma gondii* and herpes simplex virus type I, since fibroblasts are physiologically important for their infection. Additionally, these native cells stop proliferating when confluency is reached; therefore, they do not overgrow pathogen-infected cells and the proliferation of pathogens is well detectable. It was tested whether IFN-γ-induced IDO activity in the cells was able to inhibit the growth of these pathogens under normoxia (20% O_2_), intermediate oxygen conditions (5–10% O_2_) or hypoxia (1–3% O_2_).

The facultative anaerobic bacterium *Staphylococcus aureus* was able to grow in cell culture supernatants of unstimulated cells irrespective of the prevailing oxygen concentration that were used during the stimulation period. As expected bacterial growth was inhibited by IFN-γ pre-stimulated cells that had been incubated under normoxia. The lower oxygen concentration of 1% O_2_ allowed growth of Staphylococci not only in supernatants of unstimulated cells but also in supernatants of IFN-γ pre-stimulated cells indicating that the reduced IDO activity had abolished antibacterial effects (Fig. 4A). To reveal the magnitude of the IFN-γ-induced antibacterial effect, bacterial growth was determined by measuring colony forming units. Figure 4B indicates that under normoxia IFN-γ has a more then tenfold higher antibacterial activity. Comparable results were obtained for 86HG39 cells or HeLa cells (data not shown).

Figure 4C shows that the intracellular parasite *Toxoplasma gondii* proliferated well in HFF cells and, as expected, Toxoplasma growth was inhibited by IFN-γ pre-stimulation. Moreover, a detailed study revealed that this significant growth inhibition was detected when the cells were cultured in the presence of 20% O_2_, 10% O_2_ and 5% O_2_, indicating that normoxic or intermediate oxygen conditions were needed for an efficient defense against Toxoplasma. In contrast, oxygen levels below 3% O_2_ were not sufficient for parasite growth inhibition. The addition of L-tryptophan to IFN-γ-stimulated cells together with the parasites rescued Toxoplasma growth in all groups displaying that IDO-mediated tryptophan degradation was the underlying antiparasitic mechanism.

In a last set of experiments we also found out that the replication of herpes simplex virus type 1 (HSV1) was significantly inhibited in IFN-γ pre-stimulated HFF cells. Again, this inhibition of replication could only be detected when the cells were incubated under normoxic but not under hypoxic conditions (Fig. 4D).

In summary, the IFN-γ-induced IDO-mediated antibacterial, antiparasitic and antiviral defense was lost in human cells incubated under hypoxic oxygen conditions, demonstrating that hypoxia negatively influences the immune response in all analysed cell lines.

### Immunoregulatory Function of Indoleamine 2,3-dioxygenase is Abrogated Under Low Oxygen Concentrations

**Figure 5:**
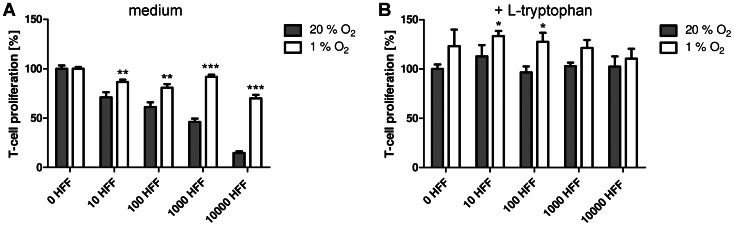
IDO-mediated immunoregulatory effect is lost under hypoxia. (A, B) Co-stimulation assay of 1.5×10^5^ PBMC and 0–10000 HFF cells/well. T-cells were activated by the anti-CD3 antibody OKT3. The T-cell proliferation in the absence (A) or presence (B) of supplemented tryptophan was determined by incorporation of [^3^H] thymidine. Data are given as T cell proliferation in % of the positive control (stimulated T cells under normoxia) +/− SEM. A significant increase of T-cell proliferation as compared to the positive normoxia control is marked with an asterisks, n = 3.

Beside its antimicrobial potency, IDO also provides immunosuppressive functions by modulating T-cell responses. As hypoxia physiologically occurs at sites of infection, T-cells frequently encounter these hypoxic regions during inflammatory processes. Therefore we were interested in the influence of hypoxia on IDO-mediated T-cell regulation. We analysed IDO-mediated T-cell regulation in the absence or presence of oxygen in an experimental setting resembling the physiological situation, without usage of synthetic IFN-γ. Therefore PBMC and HFF cells were co-cultivated for 72 h either under normoxia (20% O_2_) or hypoxia (1% O_2_). The T-cells did not proliferate unless they were activated with a monoclonal anti-CD3 antibody (data not shown). When the T-cells in the culture system were activated with OKT3 they produced IFN-γ which in turn induced IDO activity in the HFF cells. Figure 5A indicates that the T-cells proliferated in the absence or presence of oxygen; however the number of co-cultured HFF cells affected this proliferation. With increasing HFF cells in the culture system a lower proliferation rate of T-cells was detected. Importantly, this inhibitory effect was only observed under normoxia, not under hypoxia. The involvement of IDO was confirmed by additional tryptophan in the culture system, which abrogated the effect (Fig. 5B).

In summary, the hypoxia-dependent inhibition of IDO is paralleled by an enhanced T-cell proliferation since the growth of T-cells is not controlled by tryptophan starvation.

## Discussion

We investigated the expression and activity of the tryptophan-degrading enzyme IDO in the presence of different oxygen amounts *in vitro*. Under normoxic (20% O_2_) and physiologic (10% O_2_) oxygen conditions, IDO was expressed in IFN-γ-stimulated human tumour cells and fibroblasts. IDO efficiently degraded tryptophan to kynurenine which could be detected in cell culture supernatants. However, in contrast IFN-γ-activated human cells did not express IDO protein and did not produce kynurenine when they were cultured under hypoxic conditions (1% O_2_). This was in line with the findings of Roth *et al.* and Herbert *et al.* who also observed decreased IDO protein levels and a diminished IDO activity in hypoxia-treated human fallopian tube cells and monocyte-derived endothelial-like cells [10], [12]. Herbert *et al.* detected a significant reduction of IDO protein (up to 5-fold) and decrease in IDO activity (up to 100 fold) under hypoxia which was similar to our data with human tumour cells and fibroblasts (Fig. 1). The reduced IDO expression under hypoxia has been shown to result from impairment in JAK-STAT signalling [10]. We therefore evaluated JAK-STAT signalling in the different human cell lines by measurement of phosphorylated STAT1 (pSTAT1) protein amounts and found that a stimulation of the cells with IFN-γ under hypoxia resulted indeed in a reduced expression of pSTAT1. Thus, downregulation of IDO expression in human tumour cells and fibroblasts was likewise suggested to be caused by a hypoxia-induced blockade in JAK-STAT signalling. Additional experiments with JAK2 inhibitors and inhibitors of protein degradation showed no specific influence on IDO activity induced under hypoxic conditions (Fig. 2).

We proved cell survival under hypoxic conditions *in vitro* by cell viability assays and re-oxygenation studies (Fig. 3). The latter showed that IDO activity recovered when cells were incubated in normoxia for 48 h subsequent to a 72 h hypoxia treatment. Such a recovery of IDO protein and enzyme activity by reoxygenation was also observed by others [12].

The cellular response to a hypoxic environment is dependent on the hypoxia-inducible factor (HIF) protein complex. This central regulator can be activated in all mammalian cells by hypoxia where it regulates the expression of more than 100 genes included in metabolism, angiogenesis, vascular tone, cell differentiation and apoptosis [13]. The HIF complex is comprised of the constitutively expressed HIF1β subunit (also called AhR nuclear translocator; ARNT) which binds to one of two inducible α-subunits HIF1α or HIF2α [14]. Interestingly, ARNT also dimerizes with the ligand-activated aryl hydrocarbon receptor (AhR), a helix-loop-helix transcription factor that regulates cell growth, differentiation and immunological responses [15], [16]. Recently it has been shown that kynurenine, the downstream product of IDO-mediated tryptophan degradation is an endogenous ligand of AhR [5]. Given the fact that hypoxia reduces kynurenine levels by inhibiting IDO it could be possible that lower kynurenine amounts fail to activate the AhR which therefore does not translocate into the nucleus and cannot dimerize with the constitutively expressed ARNT. By this way unbound ARNT protein could be used for HIF1α binding, facilitating the cellular response to hypoxia. In this model a reduced kynurenine production by IDO would be beneficial for the cells as it enables them to adapt to the hypoxic environment.

IDO is not the only antimicrobial effector mechanism that is inhibited by hypoxia. The activities of the inducible nitric oxide synthase (iNOS or NOS2) and the phagocyte NADPH oxidase (PHOX) were impaired under hypoxic conditions as well [6]. Both enzymes are involved in the defense against bacteria in mice as iNOS- or PHOX-deficient mice had an increased susceptibility to *Staphylococcus aureus* (*S. aureus)* infections [17], [18]. Furthermore, it has been published that hypoxia inhibited mitochondrial antibacterial effector functions active against *S. aureus* and *Escherichia coli* by impairing the respiratory chain in murine macrophages [6].

However, hypoxia not only negatively but also positively influences specialized antimicrobial effector mechanisms. For example macrophages have revealed an enhanced phagocytosis and bacterial killing under hypoxia due to HIF1α activation and also the apoptosis rate of neutrophils has been diminished in a HIF1α-dependent way leading to a prolonged inflammatory response [19], [20]. Additionally, low oxygen concentrations have been shown to increase the production of the antimicrobial peptide cathelicidin within murine blood leukocytes and, although its activity was diminished by hypoxia, iNOS has been upregulated HIF1α-dependently [21], [22].

In this work we examined the consequence of a reduced IFN-γ-induced IDO expression under hypoxia on two different IDO effector functions *in vitro*. We thereby focussed not on specialized immune cells but on human fibroblasts and tumour cells that also get into contact with pathogens *in vivo* and are able to express IDO endogenously.

The first set of data describes that the human non-immune cells were no longer able to control the growth of three different pathogens prototypic for an infection with extracellular bacteria, intracellular parasites or viral infection (Fig. 4). *S. aureus* was used as model for an extracellular bacterium that causes not only skin infections but also a variety of organ-specific infections and sepsis. Animal *in vivo* studies have shown that systemic hypoxia inhibited the clearance of *S. aureus* in lung and skin infections [23], [24]. This was to some extend pinpointed to an enhanced biofilm production by the bacteria as resistance mechanism to a hypoxic environment that occurs for example in the lung of patients with cystic fibrosis [25]. Our present study now demonstrates that also an impairment of IDO could be responsible for these detrimental effects of *S. aureus* (Fig. 4A) and of other tryptophan-auxotroph bacteria *in vivo*. Nevertheless, observational cohort studies have demonstrated that an enhanced IDO activity, as detected by an increased kynurenine to tryptophan ratio in plasma, is a deleterious host response in human sepsis. A high IDO activity has been associated with decreased microvascular reactivity and T cell lymphopenia and its role as independent predictor of severe disease and case fatality has been discussed [26], [27]. Given these facts a regulation of IDO activity by hypoxia could serve as protective mechanism for the host, especially since IDO-derived bioactive tryptophan metabolites have been fatal for T cell survival, depleting overall thymocyte counts *in vitro* and *in vivo* [28].

*Toxoplasma gondii (T. gondii)* is an obligate intracellular parasite that chronically persists in healthy human beings. The infection with *T. gondii* is mostly asymptomatic; however it can cause life-threatening encephalitis, myocarditis or pneumonia in immunocompromised patients [29]. Interestingly, *T. gondii* infection of fibroblasts resulted in an up-regulation of HIF1α causing several changes in the host cell gene expression profile, thus allowing the intracellular survival of the pathogen [30], [31]. Such interplay of pathogen survival and hypoxia needs further examination in the future especially with respect to the diminished IDO-mediated antiparasitic capacities that refer to occur during hypoxic phases (Fig. 4B). Again, a reduced IDO activity could be beneficial in the case of hypoxic inflammation that is present e.g. in necrotic tissue in the centre of brain abscesses, whereas IDO activity could still mediate a protective antiparasitic effect at the border between healthy tissue and the abscess [32].

Herpes simplex virus type 1 (HSV1) was used as model for a viral infection. HSV1 infections lead to life-long persistence within the immunocompetent host but can also cause life-threatening pneumonia or encephalitis within immunocompromised patients [33], [34]. It has been shown that HSV1 replication was increased as response to low oxygen concentrations *in vitro* and that human herpes-virus 8 was reactivated under hypoxia [35], [36]. Whether this was due to a diminished IDO-mediated antiviral capacity needs to be elucidated. In fact, our data indicate that hypoxia results in an inhibition of IDO activity that allows viral growth also in IFN-γ activated human fibroblasts (Fig. 4C).

The second part of the data demonstrates that the immunoregulatory function of IDO was likewise affected in human fibroblasts by hypoxia (Fig. 5). IDO-mediated tryptophan depletion inhibited T cell proliferation in supernatants of IFN-γ pre-stimulated fibroblasts or in the presence of fibroblasts in co-stimulation assays. However, this regulatory effect on T cells was abrogated by hypoxia. Several studies have indicated that T cells underwent hypoxia-mediated and HIF-dependent cell death and therefore may only play a minor role in hypoxic tissues [13]. For example, T cells from HIF1α-deficient mice released significantly more tumor necrosis factor and IFN-γ after T cell receptor activation than wildtype T cells and mice showed an enhanced survival rate in a sepsis model [37]. Additionally also the T cell differentiation was controlled by hypoxia. Clambey *et al.* have found a robust HIF1α-dependent induction of FoxP3 and therefore an enhanced abundance of regulatory T cells under hypoxia *in vitro* and *in vivo* [38]. All these accumulating data give the hint that T cell responses are tightly regulated under hypoxic conditions, probably in order to avoid an “over-inflammation” to limit deleterious effects of inflammatory hypoxia and that in this context further analysis must be done to reveal a role of the IDO enzyme.

In conclusion our data show that an IFN-γ-induced IDO activity was inhibited under hypoxic conditions *in vitro*. Therefore IDO-mediated effector functions were lost and human tumour cells and fibroblasts were no longer able to constrain bacterial, parasitic and viral growth in the cells or in the presence of the cells. Additionally the IDO-mediated regulation of T cell proliferation was abrogated by hypoxia. Since an overwhelming IDO activity can have detrimental effects in host cells, an adjustment of IDO activity by hypoxia could serve as cellular or systemic protective mechanism.

## Materials and Methods

### Ethics Statement

This study obtained ethics approval from the ethics committee of the Medical Faculty of the Heinrich-Heine University Düsseldorf (study no. 3838). Human PBMC were generated from the blood of healthy individuals after informed and written consent.

### Cells, Media and Reagents

Human foreskin fibroblasts (HFF) (ATCC, Wesel, Germany), HeLa cells (Invitrogen, Karlsruhe, Germany) or human glioblastoma cells (86HG39) [39] were cultured in Iscove’s modified Dulbecco’s medium (IMDM) (Gibco, Grand Island, USA), supplemented with 5–10% heat-inactivated fetal calf serum (FCS). Cells were cultured in culture flasks (Costar Cambridge, USA) and split weekly in 1∶10 ratios by using trypsin/EDTA (Gibco, Grand Island, USA). Mycoplasma contamination was regularly excluded, using both culture methods and PCR.

Peripheral blood mononuclear cells (PBMC) were prepared from heparinised blood of healthy donors after density gradient centrifugation.

*Toxoplasma gondii* tachyzoites (RH strain, ATCC, Wesel, Germany) were maintained in HFF (ATCC, Wesel, Germany) in IMDM containing 5% FCS. Tachyzoites were harvested after 3 or 5 days of incubation, resuspended in PBS and used for infection experiments.

Hypoxic growth was carried out using a HERAcell 150 i CO_2_ incubator (ThermoFisher Scientific, Langenselbold, Germany) or, for Western Blot analysis, in an Invivo200 hypoxia chamber (Ruskinn) with attached flasks of nitrogen, CO_2_ and compressed air [40].

### Western Blot Analysis

5×10^5^ HFF, HeLa or 86HG39 cells were stimulated with indicated amounts of IFN-γ (30–500 U/ml) for 24 hours under normoxia (20% O_2_) or hypoxia (1% O_2_). The supernatant was discarded, 100 µL PBS containing a protease inhibitor cocktail (Roche Diagnostics GmbH, Mannheim, Germany) was added and the cells were scraped off the culture flask. Thereafter the cells were lysed by 3 freeze/thaw cycles and the cell extract was stored at −70°C after centrifugation. Proteins were separated by electrophoresis using 10% NuPAGE Novex Bis-Tris Mini gels in the appropriate electrophoresis system (Invitrogen, Karlsruhe, Germany) and semi-dry blotted on nitrocellulose membranes (CarboGlas, Schleicher & Schüll, Dassel, Germany). After blocking of the membranes in 3% (w/v) skim milk powder in TBS for 2 h at room temperature, they were incubated at 4°C overnight in the respective primary antibodies diluted in 3% (w/v) skim milk powder in TBS: anti-β-actin antibody (1∶10000, Sigma, St. Louis, USA), anti-human-IDO antibody (1∶3000, Chemicon, Hofheim, Germany), anti-human-HIF1α antibody (1∶300, BD Biosciences, Heidelberg, Germany) and anti-pSTAT1 antibody (1∶1000, Cell Signaling Technology Inc., Danvers, USA).

Thereafter the membrane was incubated for 2 h at room temperature with goat anti-mouse HRP-conjugated or goat anti-rabbit HRP-conjugated IgG (1∶10000–70000, Jackson Immuno Research Laboratories, Dianova, Hamburg, Germany), diluted in 3% (w/v) skim milk powder and 0,05% (v/v) Tween 20 in TBS. After additional washes, bands were detected by enhanced chemiluminescence (Amersham Pharmacia Biotech, Freiburg, Germany). Densitometric analysis was carried out with ImageJ software.

### Kynurenine Assay

Supernatants harvested from unstimulated or stimulated cells were analysed for their kynurenine content, using Ehrlich reagent as described before [41]. As a standard we diluted kynurenine (Sigma-Aldrich, Deisenhofen, Germany) in culture medium. For the calculation of the kynurenine content in the supernatant, linear regression and GraphPad Prism software were used.

### Inhibitor Assays

For inhibitor assays 3×10^4^ HFF cells/well were stimulated with 100 U/mL IFN-γ in the presence or absence of the proteasome inhibitor MG-132 (Sigma-Aldrich, Deisenhofen, Germany; 0,25–1 µM) [42], the sumoylation inhibitors Anancardic Acid (Sigma-Aldrich, Deisenhofen, Germany; 2,5–10 µM) or Ginkgolic Acid (Sigma-Aldrich, Deisenhofen, Germany; 2,5–10 µM) [43] or the JAK2 inhibitor BSK805 (Selleck Chemicals, Munich, Germany; 0,5–2 µM) [44]. After 72 h the kynurenine production was determined as described for the kynurenine assay.

### Determination of Bacterial Growth

For the infection experiments tryptophan-auxotroph *Staphylococcus aureus*, obtained from routine diagnostic specimens was used [45]. *S. aureus* was grown on brain heart infusion agar (Difco, Hamburg, Germany), containing 5% sheep blood and incubated at 37°C in 5% CO_2_-enriched atmosphere. For infection experiments, a 24 h old single bacterial colony was picked and resuspended in tryptophan-free RPMI 1640 (Gibco, Grand Island, USA). Bacteria were serially diluted in the same medium and 10 µl were added to 200 µl of conditioned medium corresponding to 10–100 CFU. After incubation for 16–24 h, bacterial growth was monitored using a microplate photometer (SLT Labinstruments, Crailsheim, Germany) by measuring the optical density at 620 nm. In additional experiments the CFU were determined. Therefore, the bacteria-containing cell culture media were serially diluted in PBS and 10 µl of each dilution was cultivated on Columbia blood agar plates. After 18 h resulting colonies were counted.

### Determination of Toxoplasma Growth

After pre-stimulation for 72 h at 37°C, HFF cells were infected with 2×10^4^ toxoplasma tachyzoites per well. *Toxoplasma gondii* growth was measured by the ^3^H-uracil incorporation method [46]. 48 h after infection, 0.33 µCi ^3^H-uracil were added and after additional 24 h host cells and toxoplasma were lysed by freeze thawing. ^3^H-uracil incorporation was measured using liquid scintillation spectrometry (1205 Betaplate, PerkinElmer, Jugesheim, Germany).

### Determination of Viral Replication

Infection experiments with herpes simplex virus type 1 were conducted as before [47]. In brief, pre-stimulated HFF cells were infected with 1.45×10^5^ HSV-1 copies (equal to a TCID_50_ of about 1×10^2^/well) and viral replication was monitored. Therefore a plasmid containing the amplified region was used as a standard. The amplification was carried out using an iCycler and analysed with iCycler iQ Version 3.0a (Bio-Rad, München, Germany).

### T Cell Proliferation Assays

T-cell immunoregulation by IDO was determined via two assays.

In the IFN-γ supplemented assay IDO activity was activated in 3×10^4^ HFF cells/well by IFN-γ (0, 60 or 120 U/mL) for 72 h under normoxia (20% O_2_) or hypoxia (1% O_2_). Then the tryptophan-depleted cell-free culture supernatant served as incubation medium for 1.5×10^5^ PBMC/well with or without supplemental tryptophan (100 µg/mL). The T-cells were activated by the anti-CD3 antibody OKT3 (1∶3000; American Type Culture Collection, Rockville, USA) again under normoxia or hypoxia for 72 h. Thereafter T-cell proliferation was determined by adding 0.2 µCi [^3^H] thymidine for 24 h using liquid scintillation spectrometry (1205 Betaplate, PerkinElmer, Jugesheim, Germany).

In the co-stimulation assay 0–10000 HFF cells and 1.5×10^5^ PBMC were incubated together in one well for 72 h. IFN-γ was produced by activated T-cells that were stimulated with the anti-CD3 antibody OKT3. The IFN-γ-dependent IDO induction in HFF lead to a degradation of tryptophan. The T-cell proliferation in the presence or absence of supplemented tryptophan was determined by adding [^3^H] thymidine for 24 h and measurement via liquid scintillation spectrometry.

### FACS Analysis

1×10^6^ HeLa cells/flask were stimulated with IFN-γ (0, 100 U/mL) under normoxic (20% O_2_), hypoxic (1% O_2_) or anoxic conditions in tryptophan-supplemented (100 µg/mL) IMDM cell culture medium for 72 h. Correct IDO induction was determined by measurement of kynurenine in the culture supernatants. Then the cells were harvested, centrifuged and resuspended in FACS buffer (PBS containing 2% FCS and 2 mM EDTA) or the respective antibody (mouse-anti-human HLA-DR no. 559866 or Isotype APC mouse IgG2aκ no. 555576, BD Biosciences, Heidelberg, Germany). After incubation for 60 min at RT in the dark, the samples were again centrifuged and the cells resuspended in FACS buffer and charged 1∶1000 with DAPI (Roche Diagnostics GmbH, Mannheim, Germany). As positive control for cell death, cells were harvested and additionally treated for 45 min with PFA [4%].

### Cell Viability Assay

Cell survival under normoxia (20% O_2_) or hypoxia (1% O_2_) was evaluated via alamarBlue cell viability reagent (Invitrogen, Karlsruhe, Germany). For the experiment different numbers of HFF, HeLa or 86HG39 cells (1×10^3^–3×10^4^) were incubated for the given time periods (24 h, 48 h or 72 h) in IMDM cell culture medium containing 5% FCS. Then 20 µl alamarBlue reagent was added to the cells. The resazurin within the alamarBlue is reduced to resofurin in living cells which is red in colour and can be determined by the absorbance at OD_570 nm_ vs. OD_600 nm_.

### Statistical Analysis

All experiments were done in duplicates (virus growth) or triplicates (all other experiments) and data are given as mean +/− SEM of a minimum of three independent experiments. For statistical analysis the unpaired t-test was used and significant differences were marked with asterisks (* = p<0.05; ** = p<0.01; *** = p<0.001). The analysis was performed with GraphPad Prism software.
